# Early localization of tissue at risk for delayed cerebral ischemia after aneurysmal subarachnoid hemorrhage: blood distribution on initial imaging vs early CT perfusion

**DOI:** 10.1007/s10143-024-02457-2

**Published:** 2024-05-17

**Authors:** Vesna Malinova, Beate Kranawetter, Sheri Tuzi, Veit Rohde, Dorothee Mielke

**Affiliations:** 1 Department of Neurosurgery, University Medical Center Göttingen, Göttingen, Germany; 2 Department of Neurosurgery, Georg-August-University, Robert-Koch-Straße 40, 37075 Göttingen, Germany

**Keywords:** Subarachnoid hemorrhage, Tissue at risk, CT perfusion

## Abstract

**Objective:**

Delayed cerebral ischemia (DCI) is a potentially reversible adverse event after aneurysmal subarachnoid hemorrhage (aSAH), when early detected and treated. Computer tomography perfusion (CTP) is used to identify the tissue at risk for DCI. In this study, the predictive power of early CTP was compared with that of blood distribution on initial CT for localization of tissue at risk for DCI.

**Methods:**

A consecutive patient cohort with aSAH treated between 2012 and 2020 was retrospectively analyzed. Blood distribution on CT was semi-quantitatively assessed with the Hijdra-score. The vessel territory with the most surrounding blood and the one with perfusion deficits on CTP performed on day 3 after ictus were considered to be at risk for DCI, respectively.

**Results:**

A total of 324 patients were included. Delayed infarction occurred in 17% (56/324) of patients. Early perfusion deficits were detected in 82% (46/56) of patients, 85% (39/46) of them developed infarction within the predicted vessel territory at risk. In 46% (25/56) a vessel territory at risk was reliably determined by the blood distribution. For the prediction of DCI, blood amount/distribution was inferior to CTP. Concerning the identification of “tissue at risk” for DCI, a combination of both methods resulted in an increase of sensitivity to 64%, positive predictive value to 58%, and negative predictive value to 92%.

**Conclusions:**

Regarding the DCI-prediction, early CTP was superior to blood amount/distribution, while a consideration of subarachnoid blood distribution may help identify the vessel territories at risk for DCI in patients without early perfusion deficits.

## Introduction

Delayed cerebral ischemia (DCI) is a serious complication of aneurysmal subarachnoid hemorrhage (aSAH) with the risk of developing infarction and compromising the neurological outcome of the patients [20]. According to current evidence, DCI is regarded as a potentially reversible adverse event following aSAH, given that it has been early detected and treated. Several factors such as microthrombosis, neuroinflammation, and cerebral vasospasm have been found to be involved in the pathophysiology of DCI [4]. The early manifestation of DCI on imaging is characterized by brain areas with disturbed cerebral perfusion, referred to as “tissue at risk”, that can progress to infarction [2]. The “tissue at risk” concept has its origin in ischemic stroke, where “tissue at risk” refers to time-sensitive hypoperfused but potentially salvageable brain tissue also known as penumbra [18, 28]. An early anticipation of tissue at high risk for delayed infarction is needed for precise planning of probe positioning for invasive monitoring in aSAH patients [23]. Pursuing this concept, computed tomography perfusion (CTP) has been implemented in imaging protocols for the acute management of aSAH patients with the goal of “tissue at risk” detection [6, 7, 14, 16–18, 22]. Since CTP is associated with a relevant radiation exposure, well-established parameters with high pre-test probability for detecting DCI on CTP are necessary for a reliable patient selection for CTP [5]. A practical risk chart including World Federation of Neurosurgical Societies (WFNS) grade, modified Fisher grade on admission, and age has been published to estimate the risk for DCI [3]. However, the discriminating power with AUC of 66% was rather low. Since “tissue at risk” for DCI is often localized within the brain region with the most surrounding blood, blood amount and blood distribution may be relevant for a reliable localization of “tissue at risk” for DCI. The aim of the study was to evaluate whether a semi-quantitative assessment of subarachnoid blood amount and distribution can correctly identify hypoperfused brain areas as detected with CTP performed within the first days after aneurysm rupture.

## Methods

### Patient population

In this observational study, a consecutive patient cohort with aSAH treated at our center between January 2012 and December 2020 was retrospectively analyzed. Baseline characteristics of the study population like age, WFNS grade, Fisher grade, location of ruptured aneurysm, and aneurysm treatment modality were extracted from the patients’ records.

### Semi-quantitative assessment of subarachnoid blood amount and distribution

**Fig. 1:**
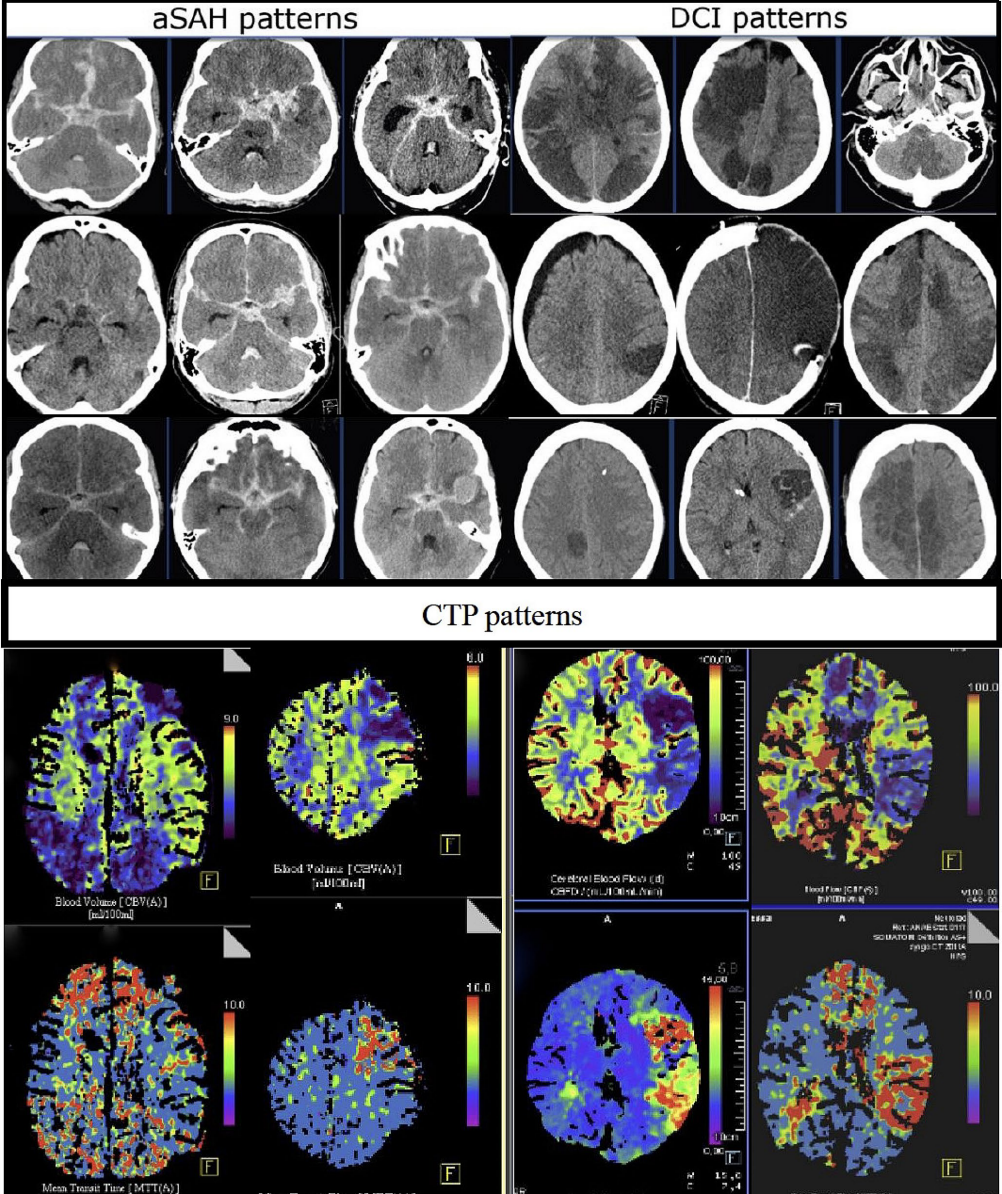
Examples of subarachnoid blood distribution compared to location of delayed infarction, and examples of perfusion deficits on CTP in patients with delayed infarctions

The subarachnoid blood amount was evaluated using the Hijdra score with assigning points (0 = no blood, 1 = small amount of blood, 2 = moderately filled with blood, 3 = filled with blood) for each subarachnoid cistern (basal and lateral Sylvian fissure, frontal interhemispheric fissure, suprasellar fissure, ambient cistern, quadrigeminal cistern), separately [8]. The subarachnoid cisterns were assigned to the cerebral arteries within these cisterns, regarding the vessel territory with the most surrounding blood as “tissue at risk” for DCI (Fig. 1).

### Assessment of perfusion deficits detected by CTP

Whole-brain CTP was performed on day 3 after the diagnosis of aSAH as part of an institutional protocol [14]. The CTP data were generated on a 128-slice multidetector CT scanner (Siemens Definition AS+, Siemens Healthcare Sector, Forchheim, Germany). The image acquisition was started 4 s after a bolus of contrast medium (Imeron 400, Bracco Imaging, Konstanz, Germany) was injected (36 ml saline solution with a flow rate of 6 ml/s). Fort the CTP, the parameters 80 kV, 200 mAs, rotation time 0.3 s, maximum pitch 0.5, and collimation 2 × 64 × 0.6 mm were used. Following CTP parameters were assessed using pre-defined cutoff values for each parameter (cerebral blood flow (CBF) < 53.93 ml/100 ml/min, time to drain (TTD) > 4.93 s, mean transit time (MTT) > 4.25 s, time to start (TTS) > 0.94 s, time to peak (TTP) > 9.28 s, and cerebral blood volume (CBV) < 3.14 ml/100 ml) [15]. Vessel territories with detected perfusion deficits on CTP were considered as “tissue at risk” for DCI.

### Primary outcome parameters

DCI was defined as the occurrence of new neurological deficits or a decrease of at least 2 points on the Glasgow Coma Scale for the duration of at least 1 h and other causes for neurological deterioration like hydrocephalus, epileptic seizures, or metabolic disturbances [26]. Delayed infarction was defined as newly diagnosed infarction on the CT scan after exclusion of treatment associated infarctions [27]. The predictive value of blood distribution on initial CT scan was compared with that of routine CTP on day 3 after ictus regarding the localization of “tissue at risk” for developing delayed infarction. The vessel territory with delayed infarction was compared with the anticipated vessel territory at risk for DCI by CTP and by the subarachnoid blood distribution.

### Statistical analysis

The statistical analyses were performed by means of the GraphPad Prism software (Version 9, GraphPad Software, San Diego, CA, USA). Descriptive statistics was used for calculation of baseline characteristics in the study population. Continuous variables are depicted as mean ± standard deviation (SD), categorical variables as frequency or percentages. Fisher’s exact test was performed to calculate odds ratios (OR), sensitivity and specificity.

## Results

### Baseline characteristics of the study population

A total of 324 patients were included. The mean age in the study population was 55 years, 64% of patients were female and 36% were male. A high WFNS grade (IV-V) had 44% of the patients, and a high Fisher grade (3–4) was found in 91% of included patients. The ruptured aneurysm was located within the anterior circulation incl. posterior communicating artery in 84% of cases, and 16% of patients had a ruptured aneurysm within the posterior circulation. The aneurysm was treated with clipping in 53% of cases and with coiling in 47% of cases. An overview of baseline characteristics is given in Table 1.

**Table 1:** Baseline characteristics

Variables	Values
**Number of patients**	324
**Mean age ± SD (range) in years**	55 ± 11
**Sex**	
- Male - Female	36% (117/324) 64% (207/324)
**WFNS grading**	
- WFNS I-III - WFNS IV-V	56% (181/324) 44% (143/324)
**Fisher grading**	
- Fisher 1–2 - Fisher 3–4	9% (29/324) 91% (295/324)
**Ruptured aneurysm location**	
- ACommA - MCA - PCommA - ACI - ACA - PCA - BA - VA - PICA	40% (130/324) 20% (65/324) 12% (39/324) 8% (26/324) 4% (13/324) 8% (26/324) 6% (19/324) 1% (3/324) 1% (3/324)
**Aneurysm treatment**	
- Clipping - Coiling	53% (172/324) 47% (152/324)

SD = standard deviation; WFNS = World Federation of Neurosurgical Societies; ACommA = anterior communicating artery; MCA = middle cerebral artery; PCommA = posterior communicating artery; ACI = internal carotid artery; ACA = anterior cerebral artery; PCA = posterior cerebral artery; BA = basilar artery; VA = vertebral artery; PICA = posterior inferior cerebellar artery

### Subarachnoid blood amount and distribution vs. perfusion deficits on CTP

**Fig. 2:**
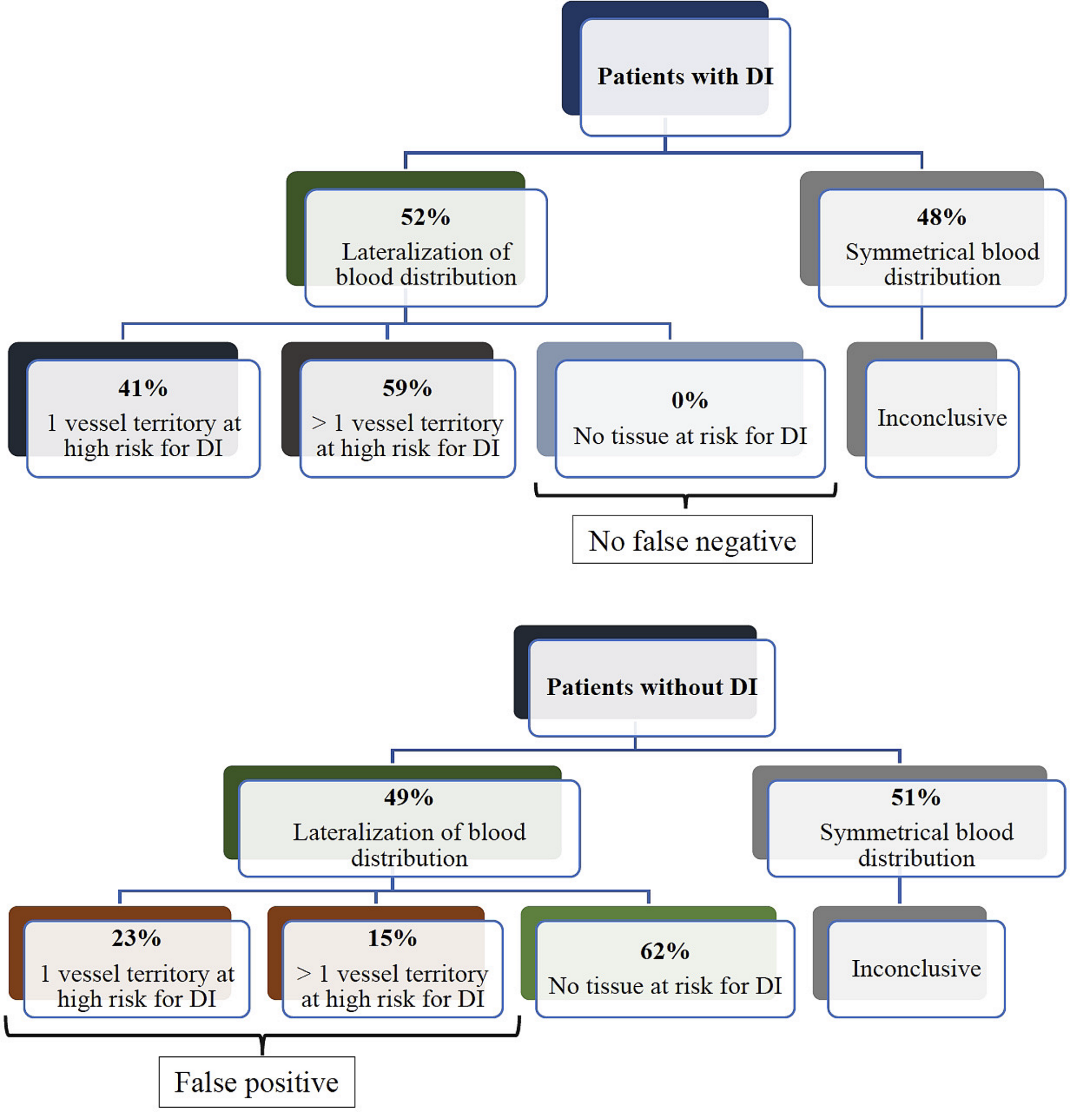
Prediction of vessel territory at risk for delayed infarction in the patient group with delayed infarction and the patient group without delayed infarction showing no false negative cases in the group with delayed infarction, but with overall 38% false positive cases in the group without delayed infarction

CTP on day 3 was performed in 26% (85/324) of patients and perfusion deficits were detected in 58% (49/85) of them. The perfusion deficits were localized within the watershed zone between the anterior and middle cerebral artery territory in 39% (19/49), within one vessel territory in 41% (20/49), in more than one vessel territory in 20% (10/49) of cases. A clear lateralization of subarachnoid blood distribution was found in 52% of the patients with delayed infarction, and in 49% in the patient group without infarction. The other half of patients in each group had a symmetrical blood distribution not allowing an identification of a vessel territory at risk for DCI (inconclusive blood distribution). In the patient group with delayed infarction and clear lateralization of blood distribution, one vessel territory was identified to be at risk for infarction in 41% and more than one vessel territory in 59% (Fig. 2). There were no false negative findings in this patient group. In the patient group without delayed infarction 62% were correctly identified to have no tissue at risk for infarction, but 23% were false positive for having one vessel territory at risk for delayed infarction and 15% for having more than one vessel territory at risk for infarction (sensitivity 100%, specificity 62%, *p* < 0.0001). Patients with more than two detected vessel territories at risk for DCI by subarachnoid blood distribution were at 5-fold higher risk for developing delayed infarction compared to their counterparts (OR 4.88, *p* = 0.01).

### Localization of delayed infarction according to blood distribuiton and perfusion deficits

**Fig. 3:**
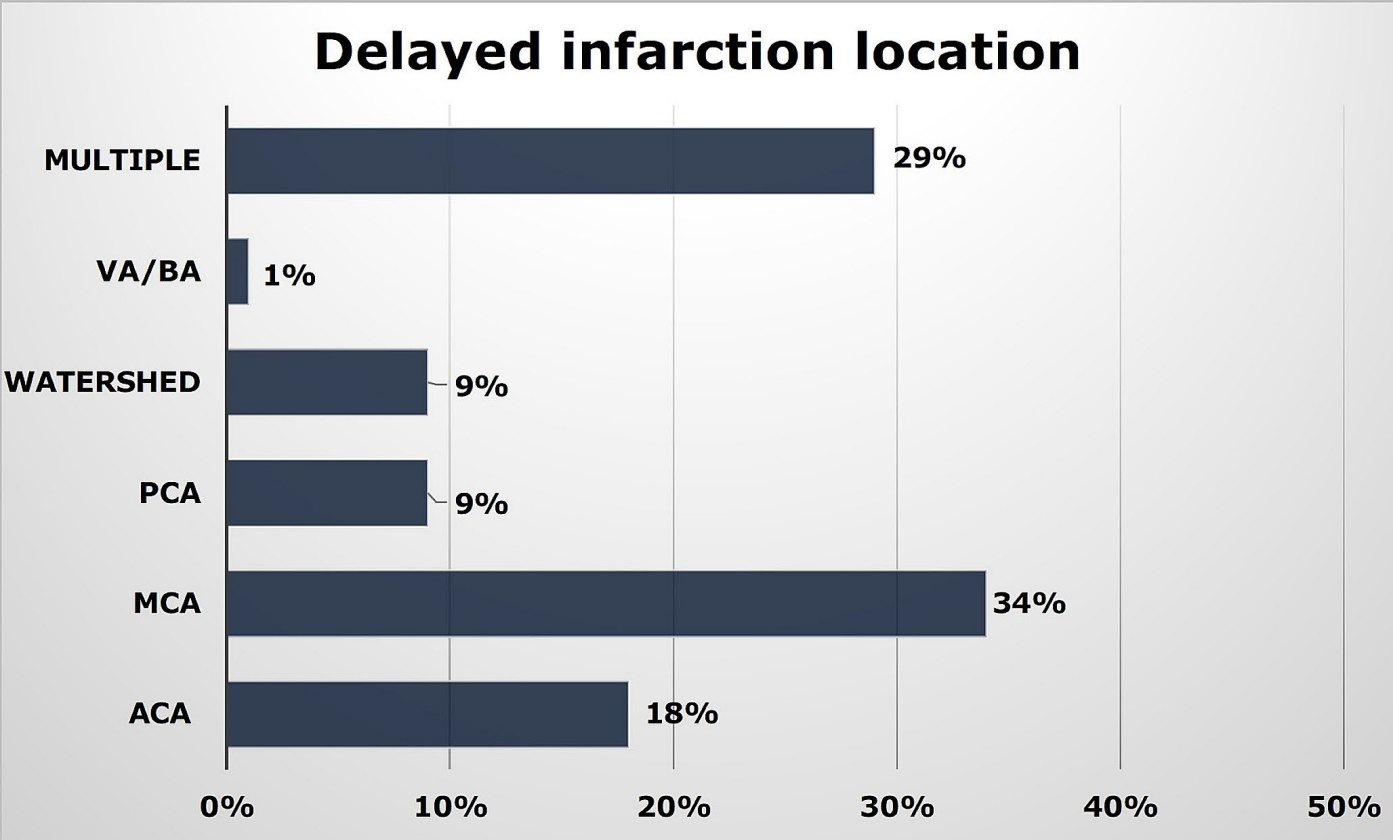
Distribution of delayed infarction location

Delayed infarction occurred in 17% (56/324) of patients. In 71% (40/56) of cases the delayed infarction affected one vessel territory and in 29% (16/56) of cases multiple vessel territories were involved (Fig. 3). Perfusion deficits were detected in 82% (46/56) of the patients with delayed infarction. An accurate prediction of the vessel territory with delayed infarction was achieved in 85% (39/46) of the patients with early perfusion deficits. In 46% (25/56) a vessel territory at risk for delayed infarction could be determined by the subarachnoid blood distribution. The vessel territory with delayed infarction was consistent with the predicted vessel territory et risk for delayed infarction in 77% (19/25). The overall detection rate of “tissue at risk” was 63% for early CTP and 36% for the subarachnoid blood distribution. A combination of both findings resulted in a detection rate of 84%. For the prediction of DCI, the blood amount/distribution was inferior to the CTP findings (Table 2). Concerning the identification of “tissue at risk” for DCI, the combination of both methods resulted in an increase of sensitivity to 64%, positive predictive value to 58%, and negative predictive value to 92% (Table 3).

**Table 2:** Prediction of delayed cerebral ischemia

Prediction of DCI	According to blood amount/distribution	According to CTP	According to both methods combined
Sensitivity (95%CI)	46% (34–59%)	94% (83–98%)	91% (81–96%)
Specificity (95%CI)	67% (61–72%)	98% (95–99%)	65% (59–70%)
Positive predictive value (95%CI)	23% (16–31%)	88% (77–95%)	35% (28–43%)
Negative predictive value (95%CI)	86% (80–90%	99% (97–100%)	97% (94–99%)
*p*-value	0.06	< 0.0001	< 0.0001

DCI = delayed cerebral ischemia, CI = confidence interval, CTP = computed tomography perfusion

**Table 3:** Identification of “tissue at risk” for delayed cerebral ischemia

Prediction of “tissue at risk” for DCI	According to blood amount/distribution	According to CTP	According to both methods combined
Sensitivity (95%CI)	30% (20–43%)	45% (32–58%)	64% (51–76%)
Specificity (95%CI)	93% (89–95%)	90% (86–93%)	90% (86–93%)
Positive predictive value (95%CI)	46% (31–62%)	48% (35–61%)	58% (46–70%)
Negative predictive value (95%CI)	87% (82–90%)	89% (84–92%)	92% (89–95%)
*p*-value	< 0.0001	< 0.0001	< 0.0001

DCI = delayed cerebral ischemia, CI = confidence interval, CTP = computed tomography perfusion

## Discussion

In contrast to ischemic stroke, where the ischemic brain area is clearly defined, disturbances of cerebral perfusion in the context of aSAH can potentially affect all brain areas, making the prediction of “tissue at risk” for DCI challenging. Delayed infarctions following aSAH can present on the CT scan as singular infarction affercting either a part of a vessel territory or the entire vessel territory, or as multiple bilateral infarctions [19, 27, 29]. Since DCI is considered a potentially modifiable risk factor for DCI, early prediction and detection of DCI has the potential of preventing delayed infarction in aSAH patients, which is one of main treatment goals in clinical practice [20, 21]. A meticulous monitoring is necessary in patients at high risk for DCI including a placement of intraparenchymal probes for continuous monitoring of brain tissue perfusion and oxygenation [1, 23, 25]. In order to meet these requirements a reliable risk stratification is needed early on after aneurysm rupture. This concept would allow an early initiation of further diagnostics and treatment in patients at high risk for DCI and prevent unnecessary routine diagnostics associated with radiation exposure in patients with low risk for DCI. The overall intracranial blood burden has been correlated with the risk for DCI and functional outcome [9–13]. In a prospective study, volumetric measurements of subarachnoid and total blood amount were found to be independent predictors of functional outcome in patients with aSAH [9]. Advanced techniques using artificial intelligence led to the development of several methods based on deep learning allowing automated volumetric analysis of blood amount [12]. A recently published study about deep learning based volumetric analysis of subarachnoid blood revealed an association of intracranial blood volume with the risk for developing hydrocephalus and with functional outcome after aSAH [12]. Automatically quantified total intracranial blood volume in a study including 369 patients confirmed that the total blood burden is an independent predictor of DCI [24]. Furthermore, volumetric analyses of subarachnoid blood in 160 patients revealed a correlation of larger blood volumes within the interhemispheric fissure and bilateral suprasellar cisterns with the incidence of DCI, but the study did not consider the location of delayed infarction [30]. Another study evaluated the relationship of vascluar territory affected by DCI with the location of ruptured aneurysm and could identify predilection sites for DCI for all aneurysm location, except for basilar artery, where multiple DCI locations were found [9]. However, the authors reported more than one vessel territory at risk for DCI in all cases, not allowing a clear ideitification oft the vessel territory the most at riks for DCI, which would be suitable for placement of monitoring probes. Another frequently used method for detection of tissue at risk for DCI represents the CTP [7, 22]. While perfusion deficits can be reliably detected at the time of infarction manifestation, early perfusion changes before the occurrence of delayed infarction are much subtle. In a previously published work of our research group, we identified CTP parameters like TTD, that demonstrated a good predictive value for early prediction of tissue at risk for DCI [15]. In this study, the predictive value of blood distribution within the subarachnoid space on intial CT scan was compared to detected perfusion deficits on early routine CTP on day 3 after ictus for a reliable identification of tissue at high risk for delayed infarction in a large consecutive aSAH-population. Subarachnoid blood distribution alone was not capable of identifying the “tissue at risk” for DCI with sufficient accuracy, since only approximately the half of patients had a clear lateralization of blood distribution. Given that a clear lateralization of blood distribution was found, subarachnoid blood distribution on admission CT scan indicated vessel territories at high risk for delayed infarction with high sensitivity, but rather low specificity. Since the overall detection rate of “tissue at risk” for delayed infarction by subarachnoid blood distribution, was lower compared to early CTP on day 3 after ictus, it may be only applied complementary to CTP as a simple risk stratification for identification of vessel territories at risk for delayed infarction, especially in patients without early perfusion deficits. Perfusion deficits detected with CTP are reflecting disturbances of cerebral microcirculation, which is the consequence of multifactorial pathophysiological processes induced by aSAH. Although, an association of higher subarachnoid blood amount with a higher incidence of ischemic complications after aSAH was indicated in previous studies, the relationship between the blood amount and distribution and the territories showing perfusion deficits on CTP has not been clarified yet. In our study, the subarachnoid blood amount and distribution exhibited a limited discrimination power concerning the identification of “tissue at risk” due to a relevant proportion (almost half of the patients) of symmetrical blood distributions found in the study population. Only in patients without perfusion deficits on the CTP and an asymmetrical blood distribution, the blood distribution may indicate the tissue at risk. Unlike the CTP, the identification of “tissue at risk” according to blood distribution led to a relevant proportion (38%) of false positive findings. Additional studies are needed to shed light on the relation of subarachnoid blood amount and distribution with the manifestation of perfusion deficits on CTP.

### Limitations of the study

The main limitation of the study is the retrospective data acquisition. The assessment of blood amount and distribution was performed semi-quantitatively, hence, we cannot exclude that volumetric analysis could have led to a different result. Especially, emerging techniques based on artificial intelligence and deep learning methods could facilitate a more precise assessment in the future.

## Conclusion

Concerning the prediction og DCI, early CTP was superior to a risk stratification according to the subarachnoid blood distribution on admission CT. However, in patients without early perfusion deficits, who have an asymmetrical blood distribution, a consideration of subarachnoid blood distribution may be supportive for identification of vessel territories at risk for DCI.

## Data Availability

All generated and analyzed data is presented in the manuscript.

## References

[1] Beck J, Raabe A, Lanfermann H, Seifert V, Weidauer S (2004) Tissue at risk concept for endovascular treatment of severe vasospasm after aneurysmal subarachnoid hemorrhage. J Neurol Neurosurg Psychiatry 75:1779–1781 15548506 10.1136/jnnp.2004.036921 PMC1738832

[2] Da Costa L, Fisher J, Mikulis DJ, Tymianski M, Fierstra J (2025) Early identification of brain tissue at risk for delayed cerebral ischemia after aneurysmal subarachnoid hemorrhage. Acta Neurochir Suppl 120:105–109 10.1007/978-3-319-04981-6_18 25366608

[3] De Rooij NK, Greving JP, Rinkel GJE, Frijns CJM (2013) Early prediction of delayed cerebral ischemia after subarachnoid hemorrhage. Development and validation of a practical risk chart. Stroke 44:1288–1294 23512975 10.1161/STROKEAHA.113.001125

[4] Dodd WS, Laurent D, Dumont AS, Hasan DM, Jabbour PM, Starke RM, Hosaka K, Polifka AJ, Hoh BL, Chalouhi N (2021) Pathophysiology of delayed cerebral ischemia after subarachnoid hemorrhage: a review. J Am Heart Assoc 10:e021845 34325514 10.1161/JAHA.121.021845 PMC8475656

[5] Döring K, Mielke D, Moerer O, Stamm G, Karsch S, Psychogios MN, Rohde V, Malinova V (2022) Radiation exposure in the acute phase after aneurysmal subarachnoid hemorrhage in the era of CT perfusion. Clin Neuroradiol 32:123–132 34505910 10.1007/s00062-021-01087-1

[6] Duan Y, Xu H, Li R, Zheng K, Hu Z, Wu N, Yang Y, Zhuge Q, Chen W (2027) Computed tomography perfusion deficits during the baseline period in aneurysmal subarachnoid hemorrhage are predictive of delayed cerebral ischemia. J Stroke Cerebrovasc Dis 26:162–168 10.1016/j.jstrokecerebrovasdis.2016.09.004 27776892

[7] Han H, Chen Y, Li R, Lin F, Lu J, Chen X, Wang S (2022) The value of early CT perfusion parameters for predicting delayed cerebral ischemia after aneurysmal subarachnoid hemorrhage: a systematic review and meta-analysis. Neurosurg Rev 45:2517–2531 35377027 10.1007/s10143-022-01779-3

[8] Hijdra A, Brouwers PJAMB, Vermeulen M, van Gijn J (1990) Grading the amount of blood on computed tomograms after subarachnoid hemorrhage. Stroke 21:1156–1161 2389295 10.1161/01.str.21.8.1156

[9] Hu P, Wu Y, Yan T, Shu L, Liu F, Xiao B, Ye M, Wu M, Lv S, Zhu X (2024) Deep learning-based quantification of total bleeding volume and its association with complications, disability, and death in patients with aneurysmal subarachnoid hemorrhage. J Neurosurg 29:1–12 10.3171/2024.1.JNS232280 38552240

[10] Hurth H, Steiner J, Birkenhauer U, Roder C, Hauser TK, Ernemann U, Tatagiba M, Ebner FH (2021) Relationship of the vascular territory affected by delayed cerebral ischemia and the location of the ruptured aneurysm in patients with aneurysmal subarachnoid hemorrhage. Neurosurg Rev 44:3479–3486 33782797 10.1007/s10143-021-01522-4 PMC8592963

[11] Ko SB, Choi HA, Carpenter AM, Helbok R, Schmidt JM, Badjatia N, Claassen J, Conolly ES, Mayer SA, Lee K (2011) Quantitative analysis of hemorrhage volume for predicting delayed cerebral ischemia after subarachnoid hemorrhage. Stroke 42:669–674 21257823 10.1161/STROKEAHA.110.600775

[12] Lagares A, Jimenez-Roldan L, Gomez PA, Munarriz PM, Castano-Leon AM, Cepeda S, Alen JF (2015) Prognostic value of the amount of bleeding after aneurysmal subarachnoid hemorrhage: a quantitative volumetric study. Neurosurgery 77:898–907 26308629 10.1227/NEU.0000000000000927

[13] Liu JJ, Raskin JS, McFarlane R, Samatham R, Cetas JS (2020) Subarachnoid hemorrhage pattern predicts acute cerebral blood flow response in the rat. Acta Neurochir Suppl 127:83–89 31407068 10.1007/978-3-030-04615-6_14

[14] Malinova V, Döring K, Psychogios MN, Rohde V, Mielke D (2021) Impact of implementing an elaborated CT perfusion protocol for aneurysmal SAH on functional outcome: CTP protocol for SAH. Am J Neuroradiol 42:1956–1961 34556476 10.3174/ajnr.A7279 PMC8583263

[15] Malinova V, Tsogkas I, Behme D, Rohde V, Psychogios MN, Mielke D (2020) Defining cutoff values for early prediction of delayed cerebral ischemia after subarachnoid hemorrhage by CT perfusion. Neurosurg Rev 43:581–587 30712134 10.1007/s10143-019-01082-8

[16] McVerry F, Dani KS, MacDougall NJJ, MacLeod MJ, Wardlaw J, Muir KW (2014) Derivation and evaluation of thresholds for core and tissue at risk of infarction using CT perfusion. J Neuroimaging 24:562–568 25039499 10.1111/jon.12134

[17] Murphy A, De Oliveira Manoel AL, Macdonald RL, Baker A, Lee TY, Marotta T, Montanera W, Aviv R, Bharatha A (2017) Changes in cerebral perfusion with induced hypertension in aneurysmal subarachnoid hemorrhage: a pilot and feasibility study. Neurocrit Care 27:3–10 28244000 10.1007/s12028-017-0379-6

[18] Phan TG, Wright PM, Markus R, Howells DW, Davis SM, Donnan GA (2002) Salvaging the ischemic penumbra: more than just reperfusion? Clin Exp Pharmacol Physiol 29:1–10 11917903 10.1046/j.1440-1681.2002.03609.x

[19] Rabinstein AA, Weigand S, Atkinson JLD, Wijdicks EFM (2005) Patterns of cerebral infarction in aneurysmal subarachnoid hemorrhage. Stroke 36:992–997 15831836 10.1161/01.STR.0000163090.59350.5a

[20] Rass V, Helbok R (2021) How to diagnose delayed cerebral ischemia and symptomatic vasospasm and prevent cerebral infarction in patients with subarachnoid hemorrhage. Curr Opin Crit Care 27:103–114 33405414 10.1097/MCC.0000000000000798

[21] Rigante L, Van Lieshout JH, Vergouwen MDI, Van Griensven CHS, Vart P, Van der Loo L, De Vries J, Vinke RS, Etminan N, Aquarius R, Gruber A, Mocco J, Welch BG, Menovsky T, Kijn CJM, Bartels RHMA, Germans MR, Hänggi D, Boogaarts HD (2022) Time trends in the risk of delayed cerebral ischemia after subarachnoid hemorrhage: a meta-analysis of randomized controlled trials. Neurosurg Focus 52:E2 35231892 10.3171/2021.12.FOCUS21473

[22] Taran S, Mandell DM, McCredie VA (2020) CT perfusion for the detection of delayed cerebral ischemia in the presence of neurologic confounders. Neurocrit Care 33:317–322 32472333 10.1007/s12028-020-01005-2 PMC7259436

[23] Tholance Y, Barcelos GK, Perret-Liaudet A, Omar E, Carrillon R, Grousson S, Lieutaud T, Dailler F, Marinesco S (2017) Placing intracerebral probes to optimize detection of delayed cerebral ischemia and allow for the prediction of patient outcome in aneurysmal subarachnoid hemorrhage. J Cereb Blood Flow Met 37:2820–2832 10.1177/0271678X16675880 PMC5536791 27798274

[24] van der Steen WE, Marquering HA, Boers AMM, Ramos LA, van den Berg R, Vergouwen MDI, Majoie CBLM, Coert BA, Vandertop WP, Verbaan D, Roos YBWEM (2019) Predicting delayed cerebral ischemia with quantified aneurysmal subarachnoid blood volume. World Neurosurg 130:e613–e619 31260850 10.1016/j.wneu.2019.06.170

[25] Veldemann M, Albanna W, Weiss M, Park S, Hoellig A, Clusmann H, Helnok R, Temel Y, Schubert GA (2021) Invasive multimodal neuromonitoring in aneurysmal subarachnoid hemorrhage: a systematic review. Stroke 52:3624–3632 34304602 10.1161/STROKEAHA.121.034633

[26] Verqouwen MD, Vermeulen M, van Gijn J, Rinkel GJ, Wijdicks FF, Muizelaar JP, Mendelow AD, Juvela S, Yonas H, Terbrugge KG, Macdonald RL, Diringer MN, Broderick JP, Dreier JP, Roos YB (2010) Definition of delayed cerebral ischemia after aneurysmal subarachnoid hemorrhage as an outcome event in clinical trials and observational studies: proposal of a multidisciplinary research group. Stroke 41:2391–2195 20798370 10.1161/STROKEAHA.110.589275

[27] Wagner M, Steinbeis P, Güresir E, Hattingen E, de Rochemont RM, Weidauer S, Berkefeld J (2013) Beyond delayed cerebral vasospasm: infarct patterns in patients with subarachnoid hemorrhage. Clin Neuroradiol 23:87–95 23010691 10.1007/s00062-012-0166-x

[28] Walther J, Kirsch EM, Hellwig L, Schmerbeck SS, Holloway PM, Buchan AM, Mergenthaler P (2023) Reinventing the penumbra – the emerging clockwork of a multi-modal mechanistic paradigm. Transl Stroke Res 14:643–666 36219377 10.1007/s12975-022-01090-9 PMC10444697

[29] Weidauer S, Lanfermann H, Raabe A, Zanella F, Seifert V, Beck J (2007) Impairment of cerebral perfusion and infarct patterns attributable to vasospasm after aneurysmal subarachnoid hemorrhage: a prospective MRI and DSA study. Stroke 38:1831–1836 17446425 10.1161/STROKEAHA.106.477976

[30] Zijlstra IA, Gathier CS, Boers AM, Marquering HA, Slooter AJ, Velthuis BK, Coert BA, Verbaan D, van den Berg R, Rinkel GJ, Majoie CB (2016) Association of automatically quantified total blood volume after aneurysmal subarachnoid hemorrhage with delayed cerebral ischemia. Am J Neuroradiol 37:1588–1593 27102313 10.3174/ajnr.A4771 PMC7984697

